# Feasibility of computerized working memory training in individuals with Huntington disease

**DOI:** 10.1371/journal.pone.0176429

**Published:** 2017-04-28

**Authors:** Mahsa Sadeghi, Emily Barlow-Krelina, Clare Gibbons, Komal T. Shaikh, Wai Lun Alan Fung, Wendy S. Meschino, Christine Till

**Affiliations:** 1 Department of Psychology, York University, Toronto, Ontario, Canada; 2 Genetics Program, North York General Hospital, Toronto, Ontario, Canada; 3 Department of Molecular Genetics, University of Toronto, Ontario, Canada; 4 Department of Psychiatry, North York General Hospital, Toronto, Ontario, Canada; 5 Department of Psychiatry, University of Toronto, Toronto, Ontario, Canada; 6 Department of Paediatrics, University of Toronto, Toronto, Ontario, Canada; University of Toronto, CANADA

## Abstract

**Objectives:**

Huntington disease (HD) is associated with a variety of cognitive deficits, with prominent difficulties in working memory (WM). WM deficits are notably compromised in early-onset and prodromal HD patients. This study aimed to determine the feasibility of a computerized WM training program (Cogmed QM), novel to the HD population.

**Methods:**

Nine patients, aged 26–62, with early stage HD underwent a 25-session (5 days/week for 5 weeks) WM training program (Cogmed QM). Training exercises involved the manipulation and storage of verbal and visuospatial information, with difficulty adapted as a function of individual performance. Neuropsychological testing was conducted before and after training, and performance on criterion WM measures (Digit Span and Spatial Span), near-transfer WM measures (Symbol Span and Auditory WM), and control measures were evaluated. Post-training interviews about patient experience were thematically analyzed using NVivo software.

**Results:**

Seven of nine patients demonstrated adherence to the training and completed all sessions within the recommended timeframe of 5 weeks. All adherent patients showed improvement on the Cogmed tasks as defined by the Improvement Index (*M* = 22.17, *SD* = 8.84, range = 13–36). All adherent patients reported that they found training helpful (*n = 7)*, and almost all felt that their memory improved (*n = 6)*. Participants also expressed that the training was difficult, sometimes frustrating, and time consuming.

**Conclusions:**

This pilot study provides support for feasibility of computerized WM training in early-stage patients with HD. Results suggest that HD patients perceive benefits of intensive WM training, though a full-scale and controlled intervention project is needed to understand the size of the effect and reliability of changes over time.

**Trial registration:**

ClinicalTrials.gov, Registry number NCT02926820

## Introduction

HD impacts motor functioning, behavioural-emotional regulation, and cognitive functioning in a stage-wise progression. Cognitive deficits in individuals with Huntington Disease (HD) may become evident well before a clinical diagnosis, with 40% of genetically confirmed individuals who do not yet meet clinical criteria reporting mild cognitive impairment [1]. Deficits in processing speed, attention, and working memory (WM) are commonly observed in early HD and have been linked to neurodegeneration of frontostratial networks [2–6]. Because of the importance of normal cognitive functioning for daily life, effective strategies that can preserve cognitive function in patients with HD are critical.

WM is a system that temporarily maintains and manipulates information for the purpose of goal-directed tasks [7]. Given the vulnerability of WM networks to frontostriatal pathology and the importance of WM for higher-level cognitive processes and daily living, an important area of rehabilitation focus for individuals with HD is to promote a functional WM system [8–9].

To date, rehabilitation studies of early-to-moderate stage HD have focused on improving motor functions such as gait and balance [10–13]. An exploratory study on the effects of multi-disciplinary rehabilitation on brain structure and cognition showed increased gray matter volume that corresponded with significant improvements in verbal learning and memory [14]. While there is a paucity of studies investigating cognitive rehabilitation in individuals with HD (in the absence of motor interventions), lifestyle factors, such as cognitive reserve [15] and educational level [16], have been suggested to significantly influence disease progression and cognitive changes in HD. Moreover, studies of R6/1 mice have shown that environmental enrichment, such as increases in sensory input and motor activity, can lead to delays of volume loss in the cerebrum, effectively delaying the onset of HD symptoms [17]. Whether it is feasible to implement a home-based computerized training program designed to enhance mental activity in individuals with HD warrants further investigation, particularly if capacity for planning and motivation are affected by the disease. Establishing feasibility of implementing a computerized cognitive training program is paramount in this population before large-scale studies addressing efficacy are performed.

We performed a pilot study in nine patients with early-stage or prodromal HD to examine the feasibility of implementing a computerized WM training program (Cogmed QM) and the qualitative experience of HD patients completing the program. Cogmed QM was chosen for its focus on WM, adaptive feedback, and systematized at-home delivery, which included weekly coaching support. Relative to other programs, Cogmed has the largest effect sizes in WM improvements following training [18], and has been shown to generalize to daily activities among child and adult populations [19–22]. We also performed an exploratory analysis examining the efficacy of the Cogmed QM program on objective neuropsychological tests.

## Methods

Research Ethics Board approval was obtained from North York General Hospital and York University in Toronto. Written informed consent was obtained from all participants at their first appointment, prior to initiation of any study procedure. This study was not registered with clinicaltrials.gov at the onset of the study because it was designed as a feasibility study and registration was not required by the study sponsor (York University). However, the trial was subsequently registered (ID number NCT02926820) at the completion of the study to conform to the requirements of PLOS ONE. The authors confirm that all ongoing and related trials for this intervention are registered. The protocol for this trial and supporting CONSORT checklist are available as supporting information; see S2 Protocol and S1 Checklist.

### Participants and recruitment

**Fig 1:**
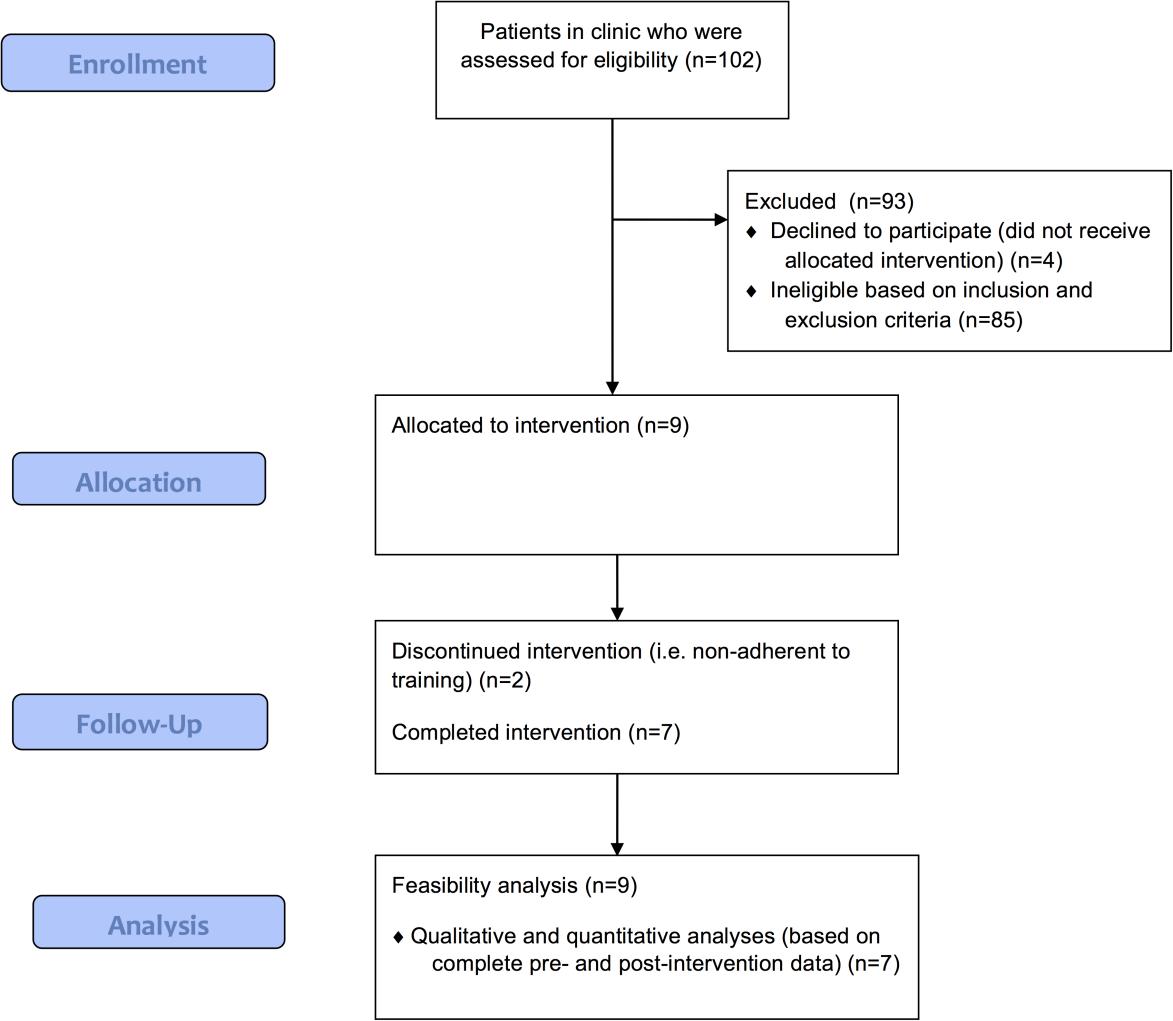
Flowchart of participant enrollment, inclusion, and involvement.

One hundred-and-two patients with pre-manifest or early-to-moderate stage HD were given recruitment letters by a genetic counsellor in the genetics clinic of North York General Hospital (NYGH), Toronto, Canada, between April 2015 and January 2016 (see Fig 1). A staff member at NYGH made follow-up calls to patients who expressed interest and met inclusion criteria based on chart review and documentation of working memory complaints on the Patient-Reported Outcomes in Cognitive Impairment (PROCOG) screener [23] (see Table 1 for exclusion and inclusion criteria). If willing to participate, clinical-demographic information was collected by phone and an appointment for baseline neuropsychological assessment was scheduled. Of 98 eligible patients, 13 expressed interest and 9 enrolled to participate (3 females; mean age = 44.25 years, SD = 9.60).

**Table 1:** Participant inclusion and exclusion criteria.

Study Participation Inclusion Criteria	Study Participation Exclusion Criteria
a. Laboratory-confirmed gene expansion of at least 36 CAG repeats b. Reported WM difficulties on the PROCOG^1^ questionnaire c. Total Functional Capacity (TFC) score of at least 3, taken from the UHDRS^2^ d. MOCA^3^ score of 19 or greater	a. History of head trauma/neurological event such as stroke b. Untreated psychiatric symptoms or substance abuse c. Visual or motor symptoms that would impede ability to complete the program and/or neuropsychological testing d. Nonfluency in English

1. The Patient-Reported Outcomes in Cognitive Impairment (PROCOG) was used as a screening instrument to confirm mild to moderate cognitive symptoms as reported by the patient.

2. The TFC score taken from the Unified Huntington’s Disease Rating Scale (UHDRS), a rating scale for clinical performance and capacity in HD which assesses motor function, cognitive function, and behavioural abnormalities.

3. The Montreal Cognitive Assessment (MOCA) is a cognitive screening test to detect mild cognitive impairment.

### Procedure

Participants underwent neuropsychological testing at either NYGH or York University at two time points: the baseline visit, and again, approximately one week following completion of training. At both time points, the psychometrist administered a 90-minute battery of questionnaires and neurocognitive tests (described below). This battery provided a comprehensive sample of WM domains, and was comprised of tests that have high validity (including use in examining cognitive functions in HD) and repeatability. The order of test administration and the psychometrist was the same for each individual and at each time point. After completing the baseline assessment, each participant met with a certified Cogmed Coach in order to become familiarized with the Cogmed QM program and to discuss the training schedule and training expectations. The individual administering the cognitive assessment was blinded to the participant’s training outcome and all testing and training was supervised by a licensed psychologist. At the follow-up assessment, a semi-structured post-training interview lasting about 15–30 minutes was conducted following the neuropsychological testing, and responses were audio-recorded and transcribed (see S1 Protocol).

Participants were instructed to complete a total of 25 sessions of the training program, typically completed over a five week period (i.e. 5 days per week), with each session lasting between 40–50 minutes per day. The program consists of 12 exercises that target visuo-spatial WM (e.g. remembering location of previously highlighted boxes) or verbal WM (e.g. remembering digits and repeating them backwards). A description of the Cogmed exercises can be found at www.cogmed.com. At each training session, participants completed 8 of the 12 exercises (order selected by the user), with 15 trials per exercise. Cogmed QM is an adaptive program, wherein task difficulty is adjusted to performance on each trial. The level of difficulty adjusts continuously and automatically, ensuring that each session provides an engaging, challenging level of WM capacity. Breaks were permitted, and encouraged, at the participants’ discretion throughout the session.

Cogmed QM was accessible through internet connection on participants’ home computers or using a laptop and internet connection provided by the research investigators. All responses were made through interacting with the program display, using a computer mouse.

A training coach made weekly phone contact throughout the training program to (i) inquire about attitude towards training, (ii) provide motivational support, and (iii) note any changes in health. The training coach accessed progress reports to verify training for each week and tracked trial-by-trial performance in order to provide individualized feedback. In addition to feedback from the training coach, the computerized program also provided both visual and verbal feedback to the participant immediately after each trial was completed by showing comparisons of previous scores and high scores and saying comments such as “way to go!” when a correct response was given.

Participants received a hospital parking pass and/or compensation for transportation, as well as a $25 gift card at each testing session.

### Neuropsychological test battery

The Wechsler Test of Adult Reading [24] was administered to estimate premorbid intelligence. Specific cognitive tests that were chosen for inclusion in this study were separated into three primary domains of interest. *Criterion measures* (i.e. those that closely resemble WM tasks in the Cogmed program) included the Wechsler Memory Scales–third edition Digit Span subtest [25] of verbal WM, as well as the Wechsler Memory Scales–third edition Spatial Span subtest [25], which assessed visuospatial WM. The second domain of interest, *near-transfer measures*, tested verbal or visuospatial WM with stimuli similar to trained tasks. Verbal WM was assessed with the Woodcock Johnson Tests of Cognitive Ability–third edition, Auditory Working Memory subtest [26], while visuospatial WM was assessed using the Symbol span subtest from the Wechsler Memory Scales–fourth edition [27]. Finally, control tasks (i.e. those that do not directly assess WM) included the Verbal Fluency subtest from the Delis-Kaplan Executive Function System [28], oral administration of the Symbol Digits Modalities Test [29], word list learning using the Hopkins Verbal Learning Test–Revised [30], and the Trail Making Test [31]. Scaled scores were calculated for all measures using available age and sex-based normative data. Double scoring was completed before computer entry of data.

In addition to performance-based tests, participants also completed questionnaires at both baseline and follow-up assessments. The self-report version of the Behaviour Rating Inventory of Executive Function [32] was administered to measure executive functioning and self-regulation in daily life as experienced by the patients. The Huntington’s Disease health-related Quality of Life questionnaire [33] was also administered to provide information related to the patient’s self-report of cognitive dysfunction and health related quality of life. Finally, the Brief Symptom Inventory (BSI) [34] was administered to assess psychological well-being and mood related symptoms.

### Cogmed training outcomes

To determine performance changes on each of the 12 Cogmed exercises, an “Improvement Index” was calculated automatically by the program by subtracting the Start Index (score on third day of training) from the Max Index (best score throughout training). The Improvement Index represents average improvement over the course of the training.

We also examined information about general performance parameters for each day of training across each exercise (i.e., trial level, successful vs. failed trials) and overall training summary statistics (i.e., total number of calendar days to complete training, mean active time per day, and mean pause time per day). Adherence was defined as completion of at least 80% of the total 25 training sessions within 40 calendar days or less. To assess tolerance, the ratio of active training time to breaks per day was examined (Cogmed guidelines suggest at most a 2:1 ratio of active training time and breaks per sessions).

### Analyses

Descriptive statistics were used to summarize clinical, demographic, cognitive, and health-related variables. Raw scores were used for all analyses. Standard scores were also calculated to explore the distribution of the sample, and to determine extreme scores. Interviews were qualitatively analyzed using QSR International’s NVivo 10 to determine common themes among participants’ experiences. Keywords were used to elicit themes that were indicative of participant attitude and motivation for training.

## Results

Clinico-demographic characteristics for all participants are shown in Table 2. Of the nine participants who were recruited, two of the patients (004 and 009) did not have a clinical diagnosis of Huntington disease, but had a positive genetic test. The median CAG length was 44 (range: 41–53); of those with a clinical diagnosis of HD, the average disease duration was 9.0 years (*SD* = 4.68, range: 2–24). The mean number of years of education completed in the sample was 14.13 (*SD* = 1.55, range = 12–16). The mean estimated IQ score (i.e. WTAR) in the sample was 103.88 (*SD* = 7.75, range = 90–117). Mood at baseline assessment, as determined by the BSI, was largely in the subclinical range (median *T* = 57.75, *SD* = 12.04, range = 42–76). Two patients fell in the clinical range of depressive symptoms (i.e. *T* scores > 70). Four of nine patients were taking prescribed antidepressant medication at time of baseline assessment. Based on the HDQoL total summary scale, patients, on average, reported a moderately high subjective quality of life (median = 73.81, *SD* = 14.83, range = 50.79–100, maximum score possible 100 signifying highest quality of life). Scores were higher on the Physical & Functional subscale (median = 86.67; *SD* = 14.17, range = 55–100) as compared with the Cognitive subscale of the HDQoL (median = 62.96; *SD* = 26.56, range = 18.52–100).

**Table 2:** Demographic and clinical characteristics for all participants (N = 9).

ID	Sex	Age	Age at Onset	Disease Duration	CAG Length (First Allele)	Education (Years)	Estimated IQ	Cognitive Concerns^1^	Mood^3^ at baseline (T score)	Clinical Dx	HDQoL Summary Scale^4^	BRIEF WM Index (T Score)
001	M	50	39	11	47	12	99	31	53	Yes	65	74
002	F	50	41	9	42	16	105	28	55	Yes	75	77
003	M	39	30	9	52	14	103	NA^2^	44	Yes	100	39
004	M	40	NA	NA	43	14	107	21	71	No	65	58
005	M	41	31	10	43	12	108	39	76	Yes	50	79
006	F	32	27	5	53	16	102	19	57	Yes	73	57
007	F	62	52	10	41	15	117	19	42	Yes	84	56
008	M	30	28	2	49	14	90	27	64	Yes	73	64
009	M	26	NA	NA	44	12	98	16	60	No	91	49

1. Patient-reported cognitive impairment using the PROCOG questionnaire. The PROCOG is a 20-item questionnaire that uses a 5-point Likert scale to allow subjective ratings of cognitive impairment. All patients’ ratings fell in the mild-moderate range of impairment.

2. This patient was not given the PROCOG for completion, in error. Cognitive complaints were confirmed at time of recruitment through an interview with hospital staff.

3. Determined by the Brief Symptom Inventory (BSI) Depression Scale. Median follow-up scores for adherent patients was *T* = 50.

4. Based on the Huntington’s Disease Quality of Life total summary scale, reflecting subjective quality of life using subscales of cognitive, physical, social, and mood functioning. Maximum possible score of 100 signifies highest quality of life.

### Adherence and tolerance

**Fig 2:**
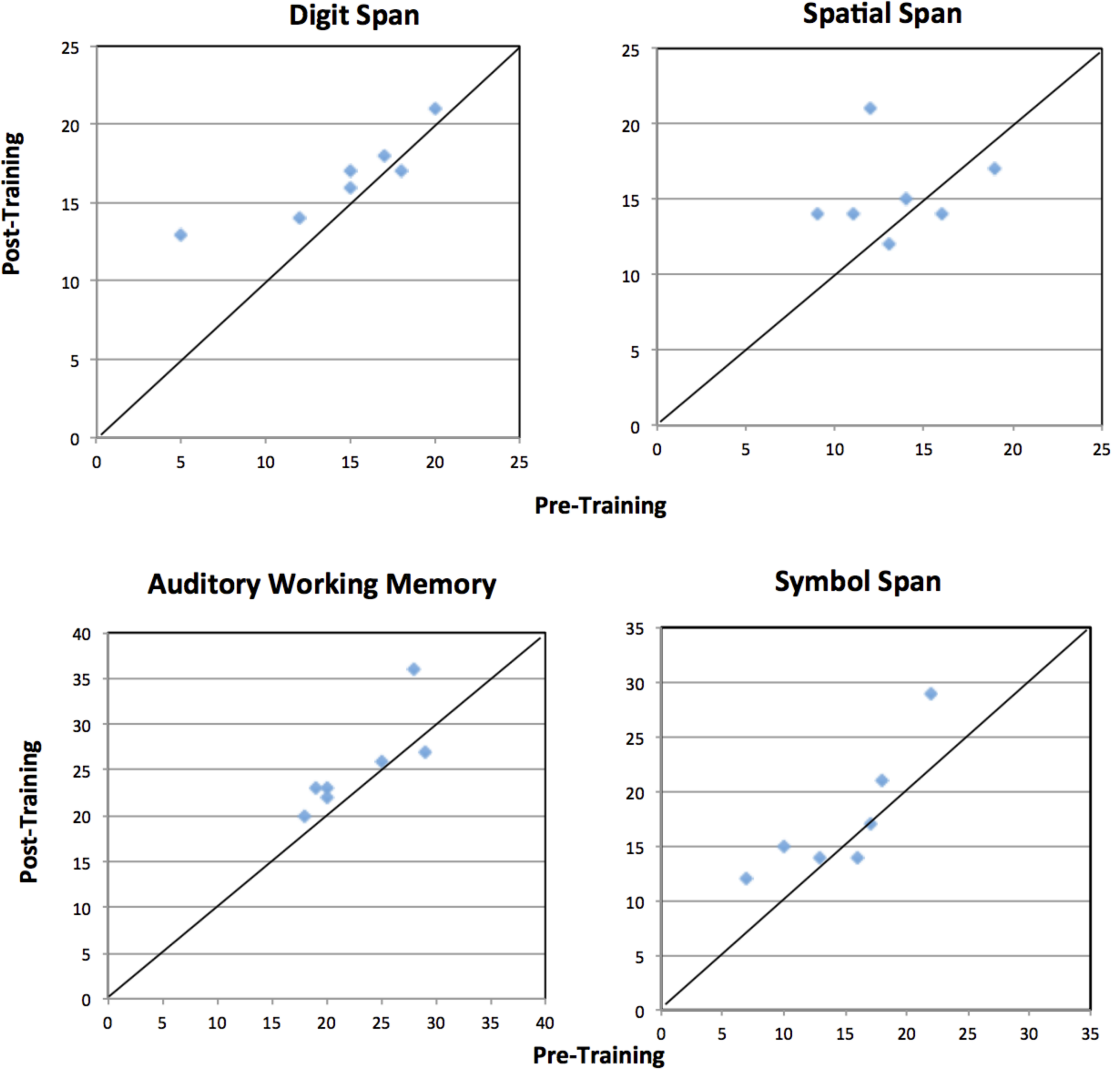
Neuropsychological data (raw scores; pre- and post-training) at the individual level plotted.

Of the nine participants recruited for the study, seven (two females) were adherent to training. All participants who attempted the training program (n = 8) were tolerant to training (see Table 3). All adherent participants showed improvement on the Cogmed tasks as defined by the Improvement Index (a measure generated by the training program to indicate extent of change on training tasks) (*M* = 22.17, *SD* = 8.84, range = 13–36). One non-adherent individual completed 9 training sessions in 28 days, and the other did not complete any training sessions. Both non-adherent participants did not return for follow-up neuropsychological assessment or post-training interview. Mean scores for each measure at baseline and follow-up testing are listed in Table 4. Individual change scores were visualized using Brinley plots (see Fig 2). Scores plotted above the diagonal line indicate better performance at follow-up.

**Table 3:** Feasibility outcomes on Cogmed QM for all participants (N = 9).

ID	Adherence (yes/no)	Tolerance (2:1 ratio of breaks/active time per session)	No. sessions completed	Length of training period (calendar days)	Mean active time per day (minutes)	Improvement Index^1^
001	Yes	Tolerant	25	34	41	13
002	Yes	Tolerant	25	34	39	29
003	Yes	Tolerant	25	43	42	15
004	No	—	0	0	0	—
005	Yes	Tolerant	24	37	31	36
006	No	Tolerant	9	33	47	3
007	Yes	Tolerant	25	34	40	22
008	Yes	Tolerant	25	38	44	8
009	Yes	Tolerant	25	33	30	24

^*1*^*Index improvement* is calculated by the Cogmed QM program by subtracting the Start Index (score on first day of training) from the Max Index (best score throughout training). The improvement score represents average improvement over the course of the training.

**Table 4:** Neuropsychological outcomes for criterion, near-transfer, and far transfer tasks using raw scores.

Measure	Baseline Median (SD)	Follow-Up Median (SD)
**CRITERION**		
Digit Span	15 (4.93)	17 (2.64)
Spatial Span	13 (3.31)	14 (2.93)
**NEAR-TRANSFER**		
Auditory Working Memory	20 (5.22)	23 (5.28)
Symbol Span	16 (5.09)	15 (5.86)
**CONTROL**		
Hopkins Verbal Learning Test	18 (5.25)	23 (5.88)
Symbol Digit Modalities Test	37 (15.69)	35 (14.02)
FAS Verbal Fluency	29 (7.61)	30 (9.09)
Trail Making Test (seconds)		
Part A	42.0 (21.42)	30.5 (26.45)
Part B	112.0 (60.33)	74.0 (84.13)

### Qualitative analysis of exit interviews

All participants who completed the Cogmed program reported that it was helpful, and viewed the feedback and instructions in the program positively. Some participants (n = 4) commented on the adaptive style of the program, especially that it kept training interesting. Participants also expressed negative aspects of training, including inconsistency or inappropriateness of feedback (n = 4), task difficulty (n = 5), frustration during training (n = 4), and that it was time consuming (n = 3).

Overall, four themes were identified with several subthemes emerging from them: (1) reported change, (2) barriers to training, (3) supports/reinforcements for training, and (4) impact of training on daily life. Table 5 presents a summary of the qualitative data analyses.

**Table 5:** Qualitative patient experiences summarized by sub-themes: 1) participant-reported change, 2) barriers to training, 3) supports/reinforcements for training, and 4) impact of training on daily life). Frequency for which subthemes were endorsed by the participants and sample quotes associated with each subtheme are presented.

Theme	Subtheme	n	Sample quote:
**1. Participant-reported changes**	Improvements in working memory, especially retaining and retrieving information.	6	*“I can remember numbers a lot better*.*”*
	Improvements in focus and attention during a task.	2	*“The focus was better*, *I could do better*, *like no matter what else was going on*.*”*
	Development and implementation of new strategies for learning.	4	*“You learn a lot of techniques in the training*. *Like how to remember*, *or how to memorize stuff you see or other ways of looking at stuff*. *So it stays in your mind more I guess*, *for that short period of time*. *At work I do a lot of accounting so I have to memorize some of the numbers that I counted*, *so in that way it kind of works out*. *Looking at stuff and memorizing where it went after*. *And so in that way it does help a lot*.*”*
	Increased motivation to try new tasks or efficacy in ability to complete a task.	4	*“Maybe it just gives me a little more confidence that I’m always good at remembering things*.*”*
**2. Barriers to training**	Internal barriers to training (e.g. feeling distracted, forgetting to complete training, feeling tired).	6	*“One day I missed it because it because I completely forgot and I didn’t want to do it late at night*, *so I skipped completely and I just did an extra day*.*”*
	External barriers to training (e.g. people nearby, pets).	6	*“I have tenants at my house that sometimes come up and say hi when they don’t know that I’m doing these tests*.*”*
**3. Supports / reinforcements for training**	Intrinsic supports such as a routine, schedule, or internal motivation.	6	*“Just getting it into a routine*, *doing it almost the same time*, *trying to get it done the same time so you can get it over and done with*. *And when you are fresh in the morning*.*”*
	Extrinsic supports such as an activity or snack.	6	*“After training…watch a movie*, *or go to bed*, *or go walk the dog or something*.*”*
	Use of social supports through the Cogmed coach, family, or friends.	5	*“She [the coach] was great coaching like that and giving you reassurance*.*”*
**4. Impact of training on daily life**	Comments on the minimal impediment training had on daily routine.	4	*“Doing the training didn’t impact me at all it was 35 or 40 minutes out of my day*, *so it was not really a big deal at all*. *I have a lot of time on my hands*.*”*
	Impact on social life.	2	*“It’s my girlfriend*. *Like there would have been less [time for her]*.*”*

## Discussion

We demonstrate the feasibility of an intensive WM training program (Cogmed QM) that is novel to the HD population, and describe the subjective experiences and training outcomes of these HD patients. Overall, 7 out of 9 patients (77%), all of whom reported cognitive concerns and had a general interest in participating in an intervention study, demonstrated adherence and tolerance to the training program. Notably, all adherent participants completed the program within the recommended timeframe of about 5 weeks and each individual training session was completed in less than one hour. Further, adherent individuals showed improvement on the Cogmed Improvement Index, demonstrating that repeated practice resulted in improvement on the Cogmed tasks. These results suggest that computerized WM training can be successfully completed by over three-fourths of individuals with pre-manifest to-early stage HD who report mild cognitive impairment and showed interest in the intervention. However, looking more broadly at the entire clinical population for whom this intervention was offered, fewer than 15 per cent of patients expressed interest, suggesting that this type of cognitive rehabilitation program may only be suitable for a small proportion of patients with HD.

Analysis of the post-training interview further support feasibility of the training program in this select sample of pre-to-early onset patients with HD. With respect to participant-reported change, six out of seven participants acknowledged improvements in their memory following the WM training. More specifically, participants reported that they could recall a larger span of numbers, consistent with their improvement on the Cogmed Improvement Index. Many individuals offered examples of situations where their memory has improved, such as in the workplace. Similar observations of subjective WM change with Cogmed training have been shown in the literature [8, 19]. Subjective reports of performance improvement must be interpreted with caution, however, as they may also reflect expectancy effects of the program [35].

The most commonly reported negative aspect of the program was the difficulty of the program. Frustration was experienced by four participants, particularly as a result of the decrease in difficulty level possible in the exercises. Further, three participants disliked the length of training, particularly on days where the exercises were longest. Despite these comments, participants explained that the training program was a chance to try something new and practice skills they felt were sometimes underused (e.g. many individuals are unemployed and enjoyed having a mental activity in their routine). Almost all participants liked the feedback they were receiving during training, both from the computer program and their coach. It is likely that individualized attention given to the individuals served as motivation to continue training, particularly since having a coach has been noted as a motivating factor [36]. Indeed, five participants mentioned social support, including the coach, as good reinforcement for training.

Internal barriers to training included feeling tired or distracted, and experiencing little improvement on an exercise. Notably, participants expressed ways they attempted to change their schedule or implement a new routine to counter such barriers (e.g. using headphones, or doing training in the morning before other tasks of the day). Four individuals stated that completing training generally reinforced a healthy routine for the day, including finding time for new hobbies or simply feeling like they had a task to complete. External barriers were mainly social, such as noise made by others in the home. Overall, the qualitative data reported by our patient sample demonstrated subjective value and pointed to potential individual differences in training success that may not be captured by neuropsychological testing, yet may influence patients well-being.

There are a several limitations of the current study that limit our ability to comment on the efficacy of this intervention. First, the study lacked a no-treatment control group and the sample size was too small for drawing meaningful conclusions regarding changes on *objective*, trained (i.e. criterion) and untrained (near-transfer) measures of WM. Observed changes on the neuropsychological measures must be interpreted with caution because they may reflect practice effects or be influence by regression to the mean. Second, the absence of information on participants who declined as well as the lack of follow-up data for those who were non-adherent restricted us from providing reasons for declining participation, which may be relevant considerations for feasibility of this intervention in the HD population as a whole. With a total of nine participants, and a male majority, generalizability of findings can only be made with caution. Additionally, participants were recruited based on specific inclusion criteria, such as stage of disease progression, existing cognitive concerns, and interest in participation. Since participants were screened for their potential to complete training, there was not enough variability in the sample to assess the relationship between outcome parameters such as active training time and performance improvements. Further studies with larger sample sizes and longer-term follow-up and including HD patients at diverse stages and cognitive impairment levels, are necessary to examine whether training gains are reliable and exist in other subsets of the HD population.

The present study’s focus on a computerized cognitive rehabilitation intervention for cognitive dysfunction in HD was novel, making this study useful in establishing new directions for treatment. Using the subjective experiences of participants, the current study provided evidence that individual factors, such as attitude towards training, influence adherence and performance. Although future replications are required, the findings provide preliminary evidence of the potential for WM improvements and highlight the need for future multi-modal determinants of efficacy.

## Supporting information

S1 Protocol Post-training interview questions. (DOCX)

S2 Protocol Trial study protocol. (PDF)

S1 Checklist TREND checklist. (PDF)
