# Supplementary material for: Feasibility of computerized working memory training in individuals with Huntington disease
Source: PLoS One. 2017 Apr 28;12(4):e0176429. doi: 10.1371/journal.pone.0176429 (PMC5409057; doi:10.1371/journal.pone.0176429)
Supplement: S1 Protocol — (DOCX) [file pone.0176429.s001.docx]

1. What did you think about the training procedure?
2. What did you find helpful about the training procedure?
3. What did you find unhelpful about the training procedure?
4. Did you face any barriers during the training procedure? What were they?
5. Compared to when you started the program, did it get harder to train towards the end?
6. Did you have any supports and/or rewards to continue your training sessions? If yes, what were they? Do you think these supports/rewards helped you get through the training?
7. How did the training impact your daily routine?
8. Do you see any effects of the training in your daily life activities?
