# Supplementary material for: Feasibility of computerized working memory training in individuals with Huntington disease
Source: PLoS One. 2017 Apr 28;12(4):e0176429. doi: 10.1371/journal.pone.0176429 (PMC5409057; doi:10.1371/journal.pone.0176429)
Supplement: S2 Protocol — (PDF) [file pone.0176429.s002.pdf]

NORTH YORK GENERAL
Making a World of Difference

RESEARCH PROTOCOL / PROPOSAL

Full Study Title: Working Memory training in individuals with Huntington disease: A pilot project

Investigators:

PI: Christine Till, PhD, C.Psych
Research Scientist, NYGH; Associate Professor, Psychology, York University
Phone #: 416-736-2100 x 20776
E-mail address: ctill@yorku.ca

Co-PIs: W.L. Alan Fung, MD, ScD, FRCPC, Psychiatry, NYGH
Wendy S. Meschino, MD, FRCPC, FCCMG, Chief & Medical Director, Genetics Program, NYGH

Co-investigators: Clare Gibbons, MS, CCGC, CGC, Genetic Counselling, NYGH
Jill Rich, PhD, C.Psych, Psychology, York University
Gary Turner, PhD, C.Psych, Psychology, York University

Collaborator: Joanne Honeyford, RN, BScN, Manager, Genetics Program, NYGH

I. PRIMARY OBJECTIVES

Aim 1. To determine feasibility of cognitive training (Cogmed) in the Huntington disease population.

Aim 2. To collect pilot data examining whether cognitive training leads to improvements (near transfer) to tasks that are not specifically trained and in perceived daily functioning as reported by patients with HD.

II. BACKGROUND AND RATIONALE

Currently, no information about the treatment of cognitive dysfunction in people with Huntington's disease (HD) is available. Cognitive enrichment through intensive computerized training may reduce the burden of cognitive symptoms, maximize function for the individual, and optimize quality of life for those living with HD. The extent of this effect is unknown.

Huntington's disease (HD) is an inherited autosomal dominant neurodegenerative disease characterized by the gradual onset and progression of motor, cognitive, and psychiatric symptoms. HD has a prevalence of 5.7 per 100,000 people in North America[1] and is caused by a CAG triplet repeat expansion of the huntingtin (HTT) gene on chromosome 4. Normally, individuals have 6 to 35 CAG repeats, whereas HD patients have >35.

Cognitive dysfunction is considered to be the most debilitating aspect of the disease and can be observed even before a clinical diagnosis of HD is made on the basis of motor symptoms.[2] Mild cognitive impairment is reported in 40% of individuals who show the genetic abnormality for HD, but do not yet meet diagnostic criteria for the disorder[3]. **Impairment in working memory (WM) across both visual[4] and verbal modalities[5-8] and reduced mental processing and psychomotor speed are frequently observed**, along with other neuropsychological impairments[4-13].

WM refers to a 'mental workspace' that allows information to be temporarily stored in the course of executing a goal.[14] WM dysfunction can be explained by the frontostriatal pathology that characterizes HD[15, 16] and is observed early in the disease process[17]. These findings are significant because of the well-known involvement of WM in a wide range of cognitive processes[18], including the processing of information necessary for reasoning, learning, comprehension, and problem-solving.

The most extensive study to-date conducted in 40 patients with early-to-middle-stage HD[19] showed that an intensive, inpatient rehabilitation program was feasible, well-tolerated and associated with positive motor benefits. Similarly, a 9-month multi-disciplinary rehabilitation program conducted in 20 patients with HD[20] was well-tolerated and associated with gains in body mass and strength. However, both programs had a minimal impact on cognitive functioning, likely reflecting an insufficient intensity of patient therapy (2 hrs/month for 6 months). **More robust studies are therefore needed in order to assess the efficacy of cognitive interventions in patients with HD.**

In mouse models of HD, environmental enrichment has been shown to delay the onset of the disease in affected mice.[21, 22] Specifically, HD transgenic mice exposed to an enriched environment showed less cerebral volume loss, enhanced exploration behaviour, and delayed onset of motor symptoms.[21] In a later study[23], findings were extended to show that even *low*-level enrichment was sufficient to mediate a significant improvement in symptoms using an accelerated HD phenotype mouse model. These findings suggest that **intensive rehabilitation based on the "use-it-or-lose-it" environmental enrichment principle might have a positive impact on patients with HD.**

In people with HD, there is mounting evidence supporting the idea of *spontaneous* brain re-organization occurring early in the disease. Functional imaging studies indicate that preclinical HD (compared to age-matched controls) is associated with differences in metabolic activity in task-dependent brain regions, but, crucially, there is no difference in task performance between the two groups, despite marked striatal atrophy.[24-31] Therefore, degeneration appears to be partially overcome by rewiring existing networks (i.e. increasing information processing efficiency of a given network) and/or recruiting new regions to a given network. ***Given that cognitively challenging activity is thought to stimulate neural activity, the proposed pilot study will examine the clinical relevance of intensive cognitive training in patients with HD.***

<u>Working Memory Training</u>

WM training aims to drive learning and adaptive neuroplastic changes in an individual's neural system through the use of specifically designed and behaviourally constrained cognitive learning events that are delivered in a scalable and reproducible manner. **The goal of a WM training program is to target known neural mechanisms underlying WM impairment in order to effect clinical change**. This type of training differs from other cognitive rehabilitation approaches designed to teach explicit strategies, such as rehearsal techniques or meta-cognitive strategies, and from more general behavioural approaches, such as exercise and mindfulness-based meditation.

Several randomized controlled studies have investigated the efficacy of the Cogmed training program, a type of training software that targets WM by using an adaptive staircase method that adjusts task difficulty on a trial-by-trial basis. Studies conducted on adults with brain injury[32, 33], stroke[34] and ADHD[35] found improvements in WM tasks that were not part of the training program (i.e. near transfer effects) with benefits maintained at a 6-month follow-up.[33] WM training also led to improvements in daily functional activity and a decrease in fatigue symptoms[33, 36] as well as improved mood.[35, 36] Another study[37] reported WM training-related gains in healthy older adults (65-75 years) with the benefits maintained at 8-month follow-up on measures of fluid intelligence and processing speed. One explanation for how benefits are maintained into the long-term is that WM training serves as a catalyst for improvement in WM capacity over time. Thus, after training, an individual may participate in activities at a more advanced level than before the training; this new level of cognitive engagement allows for the continuation (or maintenance) of WM enhancement post-training.

Relative to other cognitive training programs, a recent meta-analysis[38] showed that the Cogmed program had the highest effect size (*d*) with post-training improvements documented on both verbal (*d* =1.18) and visuospatial (*d* =.86) WM outcomes on tasks that resembled, but were not

identical to the tasks used during training. **Due to the importance of WM in everyday functioning, a near-transfer effect, on its own, is of great potential value.**

Training Working Memory Capacity in Individuals with HD: Theoretical Issues

One issue to consider when evaluating the adequacy of a training program is the **level of intensity or engagement with the training program**. The effect of intensive and continuous WM training has been likened to the effect of skill learning.[39] Over the course of intensive skill learning, a cognitive mechanism may be strengthened.[40] Greater *engagement* with the intervention is thought to increase the efficacy of the program.

A related factor that should be considered when evaluating response to intervention is a patient's **baseline level of performance**. For example, it could be argued that those with more severe impairments would show greater improvements than those with mild-to-no impairment, purely on the basis of having more "room to move." Conversely, those with milder impairments may show greater benefits of training because they are better able to capitalize on existing WM resources. Findings related to how severity of impairment impacts the efficacy of cognitive rehabilitation programs are equivocal, and may depend on the type of population under study, nature of impairment, and type of rehabilitation strategy.[41-44]

Thus, as a supplementary objective, the current pilot project will gather preliminary data to examine predictors of patient adherence and training-related efficacy.

Structural and Functional Changes as a Function of Cognitive Training

Neural mechanisms underlying the effects from WM training implicate simple Hebbian learning as an explanation for how repeated activation can improve WM capacity by strengthening the synaptic connectivity between neurons in the WM network.[18] Training-induced changes in the extent of brain activity are most commonly reported in this regard. For example, using the Cogmed program, increased brain activation was observed within a widespread cortical network and shown to co-occur with improved behavioural performance in healthy young adults.[49] Other studies in healthy adults have reported structural brain changes in parietal regions following computerized cognitive training,[50, 51] attributed to increased myelination and strengthened structural connectivity.

III. HYPOTHESES

1. HD patients with dysfunctional WM are able to adhere to a five week computerized cognitive training program (Cogmed).

2a. Completion of the WM training program will lead to improvements in measures that closely resemble the training program (i.e. criterion measures) as well as those that involve WM, but differ from the trained task (i.e. near transfer measures). Cogmed training is not expected to generalize to cognitive functions that are weakly related to WM.

2b. Patients who complete WM training will report improvements in everyday functioning, including reduced symptoms of executive dysfunction.

IV. METHODS

A. **Study Design**

A pilot study is proposed in order to collect necessary pilot data for the purpose of applying for a multi-year operating grant.

B. **Participants**
Participants will include six males or females with pre-manifest or early or moderate stage manifest HD. Participant age may range between 18 to 70 years in order to sample feasibility of cognitive training across a wide range. Disease severity will be determined by the motor scale of the Unified Huntington's Disease Rating Scale (UHDRS), which is administered in clinic. If the UHDRS is not available, then classification of motor symptoms (presence/absence) will be used.

C. **Recruitment**

HD patients who meet inclusion criteria (based on chart review) will be mailed a letter about the study (see **Appendix 1**). A follow-up call will be made shortly thereafter by a RA (staff at NYGH) to determine whether the patient is interested in the study. If the patient expresses interest, then he/she will be interviewed by phone regarding basic clinical-demographic information. Note that our goal for a full research project will be to recruit an additional 24 patients for the cognitive training intervention (out of a possible clinic sample of approximately 80 eligible patients). The 6 pilot patients from the current study will be added to the proposed sample of 24 (funds are currently being requested by the PI of this grant). Participants will provide written informed consent prior to initiation of study procedure.

The following inclusion criteria will be used:

1. Pre-manifest or manifest HD patients who report WM difficulties on the Patient Reported Outcomes of Cognition (PROCOG) instrument.[1] Those with a family history of HD must have a laboratory-confirmed gene expansion-positive of at least 36 CAG repeats.
2. Total Functional Capacity (TFC)[52] score of at least 3.
3. Montreal Cognitive Assessment (MOCA) score[53] of 19 or greater.
4. Maintenance of stable medical regimen for 4 weeks prior to study initiation and considered by the patient's clinician as being able to maintain a stable regimen for the course of the study.
5. Capacity to give informed consent.

Exclusion criteria:
1. History of cerebral trauma or other CNS disease or neurological events such as seizures or stroke. **2**. Uncontrolled psychiatric symptoms. **3.** Known history of illicit drug or alcohol abuse; **4**. Non-English speaking; **5.** Visual disturbance or extensive motor symptoms (chorea) that would interfere with completing the computerized training and/or use of mouse; **6**. Concurrent participation in any other non-pharmacologic intervention program that may affect cognition.

D. **General Procedures**

Participants will be scheduled to come to NYGH (or York University, if preferred) for a baseline assessment (T1) and introduction to the training program. As part of the baseline assessment, participants will complete a 90-minute battery to assess premorbid IQ, cognitive abilities, mood, and various lifestyle factors (e.g. leisure activities, physical activity, social support, etc.). Premorbid IQ will be estimated using the North American Adult Reading Test[54], which is a quickly administered reading test that has been shown to be a valid "hold" measure in individuals with early HD.[55] Socioeconomic status will be estimated based on years of education and occupational attainment.

Upon completion of the 5-week training program, participants will return for their post-training evaluation (T2) occurring one week ± 5 days after completing the program. Participants will receive $25 at each test session.

[1] While a loss of awareness may be observed for more advanced patients, early stage HD patients are shown to accurately judge their own memory disorders and are even more accurate than ratings made by proxies, who tend to be more influenced by patients' functional decline rather than by patients' bona fide cognitive deficits.[62]

The same examiner will administer the measures at each time point in order to decrease the potential for examiner-related variance in test administration. Personnel performing the neuropsycho-logical evaluations will remain blind to group status of the patient. Training Coaches will not be involved in the testing of patients. All questions/communications will be directed through a research manager who will not see patients for interventions or assessments.

**Parameter**	**Recruit-ment** **Week 0**	**Baseline** **Week 1**	**Training** **Weeks 1-6**	**Immed. Follow up** **Week7**
Chart Review Abstraction Form – completed by RA prior to phoning patient	X			
Demographic Form – completed by phone	X			
Godin-Shepard Leisure Activity Questionnaire (5 min)		X		
NEO-FFI-3 (Personality measure) (10 min)		X		
Premorbid IQ (NAART-35) (5 min)		X		
Cognitive battery (50 minutes)		X		X
Huntington's Disease Quality of Life (HDQoL) (5 min)		X		X
Situational Motivation Scale (5 min)		X		
Brief Symptom Inventory (BSI) (10 minutes)		X		X
BRIEF (executive function behaviour questionnaire) (10 min)		X		X
Cogmed training (30 min)		X		
Cogmed outcomes and weekly coaching calls (including assessment of any adverse events)			1 2 3 4 5	
Exit interview				X
**TOTAL TIME INVOLVED FOR PATIENT**	**30 min**	**120 min**	**20 min / call**	**120 min**

WM Training Program. Cogmed QM (Pearson, 2011) is a computerized program that is run on the internet. This online, home training program is advantageous because it allows progress tracking to be verified. The program has 12 exercises of which the user completes eight per day; exercises rotate each day to provide novelty and maintain interest. The exercises tap into both verbal WM (e.g. remembering letters, digits) and visuo-spatial WM (e.g. remembering position of objects).

Participants train on the tasks for ~40 minutes per day for 25 sessions (roughly 5 days/week x 5 weeks). Training extending beyond 7 weeks is considered to be less effective because the intensity of the training may be diluted. Participants complete 15 trials per exercise (i.e. 15 x 8 =120 trials/ day). For each exercise, responses are made by clicking on displays with a computer mouse. The user chooses the order in which the exercises are completed. Breaks can be scheduled within the training session at the participant's discretion.

The adaptive version of the Cogmed program will be used. In this version, task difficulty is matched to the individual's level on a trial-by-trial basis for each task. Difficulty level for each task is adjusted automatically and continuously as the participant improves during each session so that an optimal level of challenge is maintained and participants are always engaging their WM capacity.

Immediate feedback is provided to all participants by presenting best score relative to previous scores. In addition, a Training Coach (clinical psychology graduate student supervised by a registered psychologist) will make weekly phone contact with the individual to provide support and

feedback on the basis of the results from the last 5 training days. During this weekly call, the Training Coach will ask questions related to fatigue, adverse events, and any changes to overall health status.

E. **Outcomes and Analytic Plan**

Tests were selected based on the following criteria: (i) sound psychometric properties, including high validity (i.e., shown to relate to the psychological construct under study; used in previous studies examining cognition in HD) and test-retest reliability[56], (ii) repeatable, with minimal practice effects; (iii) brief enough to permit comprehensive sampling of cognitive functioning across visual and verbal WM domains; and (iv) reduced reliance on psycho-motor speed.

Descriptive statistics will first be calculated for the clinico-demographic, cognitive, and other health-related variables to characterize the patient sample. Data will be analyzed using SPSS (SPSS Corp., Chicago, Ill, USA).

Aim 1 (feasibility). The following dependent measures will be examined:

Measure	Operational definition
**Adherence**	Number of patients who complete 25 sessions within a 6-week training period.
**Tolerance of training**	Number of minutes spent in a break as compared with active training. Participants requiring >20 minutes break for 40 minutes of training will be identified as having difficulty with tolerating the program within recommended training time. Subjective report of tolerability will also be recorded during weekly coach calls.
**Cogmed Improvement Index (CII)**	Derived by subtracting the Start Index (calculated with Day 2 and 3 results) from the Max Index (results of two best days during the training period). The mean Improvement Index for individuals aged 18 to 65 years is 29 (normal range is 15 to 41), with higher scores signifying good compliance and effort with the training.

Aim 2 (near-transfer effects and perceived differences) will be addressed by examining changes in cognitive performance (i.e., ΔT2-T1) on ***criterion*** (i.e. similar to training exercises), ***near-transfer*** (non-similar to training exercises), and ***far-transfer measures*** (i.e. not directly related to WM) as described below (and in the Appendix).

Criterion measures	***Near transfer measures***	***Far transfer measures***
Digit Span – Backward (WMS-III) will assess auditory-verbal WM. This measure requires the participant to repeat a series of digits backwards.<br><br>Spatial Span – Forward (WMS-III) will assess visual-spatial WM capacity. The examiner taps irregularly arranged cubes on a board in a non-sequential order. The patient is required to repeat the sequence of taps in forward order.	Auditory-Verbal WM (WJ-III). After hearing a series of digits and words, participants must re-order the series, starting with the words and then providing digits in order.<br><br>Symbol Span (WMS-IV) is a visual analogue to 'digit span'. Patients are asked to remember a sequence of abstract designs and then to select the design from foils in the correct sequence.	Symbol Digits Modalities Test (SDMT; oral administration) assesses visuo-perceptual scanning speed and attention.<br><br>Hopkins Verbal Learning Test assesses learning of a word list.<br><br>Trail Making Test (parts A & B) assesses psycho-motor speed visuo-spatial attention, and flexibility.<br><br>Phonetic verbal fluency (DKEFS) – measure of phonemic fluency. Score reflects number of correct words produced across three 1-minute trials.
Perceived differences will be addressed by evaluating change in patient-reported:		
• **Quality of Life** assessed using the Huntington's Disease Quality of Life (HDQoL) scale[57]		

- **Mood** assessed using the Brief Symptom Inventory (BSI)
- **Executive dysfunction** using Behavior Rating Inventory of Executive Dysfunction – Adult[59]

Analysis of covariance (ANCOVA) will be used to analyze performance changes on each type of measure, controlling for baseline (T1) outcome measure scores. Standard model checking will be conducted to ensure adequate model fit. Estimates of the treatment effect will be summarized using results from the adjusted ANCOVA model, along with 95% confidence intervals and standardized effect sizes. Supplementary analyses will examine how cognitive change scores associate with tolerance (i.e., time on-task) and improvement on the Cogmed training program.

F. Available Resources

The Genetics Clinic at NYGH, a multidisciplinary clinic established in 1994, is one of the few institutions in Canada to care for a large number of patients with HD. The clinic has a **detailed clinical database** consisting of over 200 manifest and pre-manifest patients, which provides a sufficient patient population to obtain the proposed sample size of 24 HD patients. This established database will also ensure access to high-quality clinical and demographic data for valid review of patient histories. Moreover, our multi-disciplinary, **investigative team has the necessary expertise** spanning genetics, neuropsychology, psychiatry, and rehabilitation to provide the basis for neuro-rehabilitative care and research in HD. Members of our team are already participating in clinical research and pharmacologic intervention trials in this population.

Expected Scholarly Output/Outcome (e.g. presentation at conference, publication, or "real-world" impact of innovation).

Findings from this study will be used to collect necessary pilot data that can be used in future grant applications that will be focused on addressing a major knowledge gap identified by knowledge users in the field of HD – that is, *can cognitive dysfunction be treated using computerized cognitive training?*

The focus of the study is also well aligned with Ontario Health Care's emphasis on using technology to enhance the delivery of patient care. Important 'real world' outcomes in the study will include an understanding of patients' tolerance for intensive cognitive training, knowledge regarding patient characteristics that may influence outcomes, and ultimately, whether computerized cognitive training may provide prosthetic benefit in the face of neurodegenerative-related cognitive decline. We anticipate the results of the study will also be of importance to other research groups examining non-pharmacologic methods to prevent cognitive decline in elderly and neurologic populations.

Regarding expected scholarly output, target audiences that we plan to reach with our dissemination plan include the HD Society of Canada as well as clinicians and researchers in the field of HD and rehabilitation. Throughout the research process, we anticipate numerous scholarly translation activities, including publication of key findings in at least two peer-reviewed publications as well as presentations at national and international conferences related to HD and neurorehabilitation. We will target journals such as Archives of Clinical Neuropsychology, Journal of Huntington's Disease, and Movement Disorders. Finally, presentations about the results of this study will be prepared for service providers, community agencies, grand rounds at NYGH, as well as seminars at York University.

One page, single-spaced (Arial 11 pt font). Must be written to be understandable to a scientifically educated but not necessarily expert audience. A brief description of the expected significance or impact of the research/innovation and an overview of how results will be disseminated

Despite the identification of the gene responsible for Huntington's disease (HD) over 20 years ago, there are no effective treatments available to patients to modify disease progression and improve their quality of life. The high prevalence of cognitive impairment in HD and its negative and progressive impact on patients' everyday life indicate a clear need for feasible interventions that can *delay* cognitive decline in this population.

The proposed pilot study will determine feasibility in conducting a WM training program in prodromal or early-to-moderate stage HD patients among whom treatment effects are more likely to be observed. In contrast to other studies that have used less intensive programs for the treatment of cognitive problems in HD patients, the proposed study will use an *intensive* computerized training program that is *individualized* for each patient's level of functioning and can *adapt* the level of challenge in order to promote effective learning while a training coach carefully monitors progress on a weekly basis.

The multi-modal outcomes (i.e. examination of near- and far-transfer effects and inclusion of functional outcome measures to explore whether the intervention generates improvement in 'real world' functioning) is important in our pilot phase in order to determine the sensitivity of our outcome measures.

The need for effective neurorehabilitation to minimize the onset of cognitive decline in patients with HD is critical. We argue that more research examining outcomes of cognitive training will lead to more effective interventions. If we can boost brain plasticity to stave off decline, then therapeutic programming can be recommended and further developed to ensure that therapy is delivered within an optimal time frame and with sufficient intensity in order to maintain healthy cognitive functioning for as long as possible. A direct application of our findings will be the implementation of early intervention to maintain functional abilities in HD patients.

Future studies can use the findings from this study to test the stability of the intervention effect into the longer-term (i.e. five-month outcomes) and to refine the neuropsychological intervention (i.e. inclusion of regular 'booster' sessions) and/or criteria for assessing outcomes, as needed. If cognitive training reveals a change in behavioural performance, future studies will be designed to understand the underlying neural mechanisms that drive the observed changes. This type of information could be used to inform future clinical trials where there is a need to use cognitive and neuroimaging outcomes that are relevant to treatment targets.

Signature of Applicant

Appendix. Investigative Team.

Principal Investigator:
Dr. Christine Till is an associate professor of psychology at York University and a Research Scientist at North York General Hospital. Her research combines rigorous clinical and experimental techniques with biologically-based methodologies, including electrophysiology and state-of-the-art neuroimaging techniques. She has expertise in examining the neural basis for cognitive impairment in individuals with diffuse brain injury across the lifespan. She serves as the lead investigator for studies related to cognitive and behavioural, outcomes in the Canadian Pediatric Acquired Demyelination Network and teaches in a clinical psychology graduate program. As a clinical neuropsychologist, she will be responsible for all clinical issues related to cognitive testing and working memory training. Dr. Till will be responsible for supervising the research assistant and psychometrist's activities. Finally, Dr. Till will assist with the statistical analyses, dissemination of findings and preparation of manuscripts for publication.

Co-Applicants:
**Dr. Gary Turner is an assistant professor of Psychology at York University and an Adjunct Scientist at Sunnybrook Hospital. He is a clinical neuropsychologist with expertise in the assessment of executive functions, cognitive rehabilitation, and in functional neuroimaging. He is experienced in conducting placebo-controlled behavioural intervention trials with patient populations and will assist with the logistical issues specific to the proposed intervention and planned analyses.

Dr. Wendy S. Meschino is the Chief of Genetics and Program Medical Director at North York General Hospital. Her area of expertise is in the communication of genetic information to health-care providers, patients and the public, with a particular expertise in Huntington's disease. Dr. Meschino will provide a role in determining HD diagnosis and comorbidities and will partake in data interpretation and final manuscript preparation.

Dr. W. L. Alan Fung, a psychiatrist, is the Medical Director of Research (Mental Health Program) at North York General Hospital with expertise in the management of psychiatric issues in individuals with Huntington's disease. He is also experienced in conducting clinical research and specifically in examining patient-related outcomes in populations with neurodegenerative disease. Dr. Fung will provide a role in determining suitability of candidates for the current study, and will partake in data interpretation and final manuscript preparation.

Clare Gibbons, MS is a genetic counselor at North York General Hospital with considerable clinical and research expertise related to Huntington's disease. She will assist with patient recruitment, chart review, and will partake in data interpretation and final manuscript preparation.

Dr. Jill Rich is an associate professor of Psychology at York University and a clinical neuropsychologist. She has considerable experience conducting behavioural research in Huntington's disease, as well as other aging and clinical populations who have or are at risk of cognitive decline. Dr. Rich will assist with the supervision of trainees involved in the project and will provide assistance with data interpretation and final manuscript preparation.

Collaborator: Joanne Honeyford, who serves as the Manager of the Genetics Program at NYGH will assist with overseeing patient recruitment and supervising the Research Assistant at NYGH.

References

1. Pringsheim T, Wiltshire K, Day L, Dykeman J, Steeves T, Jette N. The incidence and prevalence of huntington's disease: A systematic review and meta-analysis. Mov Disord. 2012; 27(9):1083-1091.

2. Paulsen JS, Langbehn DR, Stout JC, et al. Detection of huntington's disease decades before diagnosis: The predict-HD study. J Neurol Neurosurg Psychiatry. 2008; 79(8):874-880.

3. Duff K, Paulsen J, Mills J, et al. Mild cognitive impairment in prediagnosed huntington disease. Neurology. 2010; 75(6):500-507.

4. Dumas EM, Say MJ, Jones R, et al. Visual working memory impairment in premanifest gene-carriers and early huntington's disease. J Huntingtons Dis. 2012; 1(1):97-106.

5. Papp KV, Snyder PJ, Mills JA, et al. Measuring executive dysfunction longitudinally and in relation to genetic burden, brain volumetrics, and depression in prodromal huntington disease. Arch Clin Neuropsychol. 2013; 28(2):156-168.

6. You SC, Geschwind MD, Sha SJ, et al. Executive functions in premanifest huntington's disease. Mov Disord. 2014; 29(3):405-409.

7. Harrington DL, Liu D, Smith MM, et al. Neuroanatomical correlates of cognitive functioning in prodromal huntington disease. Brain Behav. 2014; 4(1):29-40.

8. Duff K, Paulsen JS, Beglinger LJ, et al. "Frontal" behaviors before the diagnosis of huntington's disease and their relationship to markers of disease progression: Evidence of early lack of awareness. J Neuropsychiatry Clin Neurosci. 2010; 22(2):196-207.

9. Hanes KR, Andrewes DG, Smith DJ, Pantelis C. A brief assessment of executive control dysfunction: Discriminant validity and homogeneity of planning, set shift, and fluency measures. Arch Clin Neuropsychol. 1996; 11(3):185-191.

10. Paulsen JS, Como P, Rey G, et al. The clinical utility of the stroop test in a multicenter study of Huntington’s disease. . Journal of the International Neuropsychological Society. 1996; 2(35):2.

11. Watkins LH, Rogers RD, Lawrence AD, Sahakian BJ, Rosser AE, Robbins TW. Impaired planning but intact decision making in early huntington's disease: Implications for specific fronto-striatal pathology. Neuropsychologia. 2000; 38(8):1112-1125.

12. Rich JB, Troyer AK, Bylsma FW, Brandt J. Longitudinal analysis of phonemic clustering and switching during word-list generation in huntington's disease. Neuropsychology. 1999; 13(4):525-531.

13. Rich JB, Campodonico JR, Rothlind J, Bylsma FW, Brandt J. Perseverations during paired-associate learning in huntington's disease. J Clin Exp Neuropsychol. 1997; 19(2):191-203.

14. Baddeley A. Working memory. Science. 1992; 255(5044):556-559.

15. Aylward EH. Change in MRI striatal volumes as a biomarker in preclinical huntington's disease. Brain Res Bull. 2007; 72(2-3):152-158.

16. Hobbs NZ, Pedrick AV, Say MJ, et al. The structural involvement of the cingulate cortex in premanifest and early huntington's disease. Mov Disord. 2011; 26(9):1684-1690.

17. Nopoulos P, Magnotta VA, Mikos A, Paulson H, Andreasen NC, Paulsen JS. Morphology of the cerebral cortex in preclinical huntington's disease. Am J Psychiatry. 2007; 164(9):1428-1434.

18. Klingberg T. Training and plasticity of working memory. Trends Cogn Sci. 2010; 14(7):317-324.

19. Zinzi P, Salmaso D, De Grandis R, et al. Effects of an intensive rehabilitation programme on patients with huntington's disease: A pilot study. Clin Rehabil. 2007; 21(7):603-613.

20. Thompson JA, Cruickshank TM, Penailillo LE, et al. The effects of multidisciplinary rehabilitation in patients with early-to-middle-stage huntington's disease: A pilot study. European Journal of Neurology. 2013; 20(9):1325-1329.

21. van Dellen A, Blakemore C, Deacon R, York D, Hannan AJ. Delaying the onset of huntington's in mice. Nature. 2000; 404(6779):721-722.

22. Spires TL, Grote HE, Varshney NK, et al. Environmental enrichment rescues protein deficits in a mouse model of huntington's disease, indicating a possible disease mechanism. J Neurosci. 2004; 24(9):2270-2276.

23. Hockly E, Cordery PM, Woodman B, et al. Environmental enrichment slows disease progression in R6/2 huntington's disease mice. Ann Neurol. 2002; 51(2):235-242.

24. Wolf RC, Vasic N, Schonfeldt-Lecuona C, Landwehrmeyer GB, Ecker D. Dorsolateral prefrontal cortex dysfunction in presymptomatic huntington's disease: Evidence from event-related fMRI. Brain. 2007; 130(Pt 11):2845-2857.

25. Wolf RC, Sambataro F, Vasic N, Schonfeldt-Lecuona C, Ecker D, Landwehrmeyer B. Aberrant connectivity of lateral prefrontal networks in presymptomatic huntington's disease. Exp Neurol. 2008; 213(1):137-144.

26. Wolf RC, Sambataro F, Vasic N, et al. Abnormal resting-state connectivity of motor and cognitive networks in early manifest huntington's disease. Psychol Med. 2014; 44(15):3341-3356.

27. Novak MJ, Warren JD, Henley SM, Draganski B, Frackowiak RS, Tabrizi SJ. Altered brain mechanisms of emotion processing in pre-manifest huntington's disease. Brain. 2012; 135(Pt 4):1165-1179.

28. Georgiou-Karistianis N, Poudel GR, Dominguez DJF, et al. Functional and connectivity changes during working memory in huntington's disease: 18 month longitudinal data from the IMAGE-HD study. Brain Cogn. 2013; 83(1):80-91.

29. Ferraro S, Nanetti L, Piacentini S, et al. Frontal cortex BOLD signal changes in pre-manifest huntington disease: A possible fMRI biomarker. Neurology. 2014; 83(1):65-72.

30. Odish OF, van den Berg-Huysmans AA, van den Bogaard SJ, et al. Longitudinal resting state fMRI analysis in healthy controls and premanifest huntington's disease gene carriers: A three-year follow-up study. Hum Brain Mapp. 2014; .

31. Rao JA, Harrington DL, Durgerian S, et al. Disruption of response inhibition circuits in prodromal huntington disease. Cortex. 2014; 58:72-85.

32. Akerlund E, Esbjornsson E, Sunnerhagen KS, Bjorkdahl A. Can computerized working memory training improve impaired working memory, cognition and psychological health? Brain Inj. 2013; 27(13-14):1649-1657.

33. Johansson B, Tornmalm M. Working memory training for patients with acquired brain injury: Effects in daily life. Scand J Occup Ther. 2012; 19(2):176-183.

34. Westerberg H, Jacobaeus H, Hirvikoski T, et al. Computerized working memory training after stroke--a pilot study. Brain Inj. 2007; 21(1):21-29.

35. Gropper RJ, Gotlieb H, Kronitz R, Tannock R. Working memory training in college students with ADHD or LD. J Atten Disord. 2014; 18(4):331-345.

36. Bjorkdahl A, Akerlund E, Svensson S, Esbjornsson E. A randomized study of computerized working memory training and effects on functioning in everyday life for patients with brain injury. Brain Inj. 2013; 27(13-14):1658-1665.

37. Borella E, Carretti B, Riboldi F, De Beni R. Working memory training in older adults: Evidence of transfer and maintenance effects. Psychol Aging. 2010; 25(4):767-778.

38. Melby-Lervag M, Hulme C. Is working memory training effective? A meta-analytic review. Dev Psychol. 2013; 49(2):270-291.

39. Arnett PA, Rao SM, Bernardin L, Grafman J, Yetkin FZ, Lobeck L. Relationship between frontal lobe lesions and wisconsin card sorting test performance in patients with multiple sclerosis. Neurology. 1994; 44(3):420-425.

40. Audoin B, Ibarrola D, Au Duong MV, et al. Functional MRI study of PASAT in normal subjects. MAGMA. 2005; 18(2):96-102.

41. Goverover Y, Chiaravalloti ND, Deluca J. Task meaningfulness and degree of cognitive impairment: Do they affect self-generated learning in persons with multiple sclerosis? Neuropsychol Rehabil. 2014; 24(2):155-171.

42. Chiaravalloti N, Hillary F, Ricker J, et al. Cerebral activation patterns during working memory performance in multiple sclerosis using FMRI. J Clin Exp Neuropsychol. 2005; 27(1):33-54.

43. Barrett AM, Crucian GP, Schwartz RL, Heilman KM. Testing memory for self-generated items in dementia: Method makes a difference. Neurology. 2000; 54(6):1258-1264.

44. Gonzalez-Nosti M, Arango-Lasprilla JC, Cuetos F. The generation effect in patients with mild cognitive impairment. Am J Alzheimers Dis Other Demen. 2010; 25(7):576-584.

45. Aylward EH. Magnetic resonance imaging striatal volumes: A biomarker for clinical trials in huntington's disease. Mov Disord. 2014; 29(11):1429-1433.

46. Novak MJ, Seunarine KK, Gibbard CR, et al. White matter integrity in premanifest and early huntington's disease is related to caudate loss and disease progression. Cortex. 2014; 52:98-112.

47. Scahill RI, Hobbs NZ, Say MJ, et al. Clinical impairment in premanifest and early huntington's disease is associated with regionally specific atrophy. Hum Brain Mapp. 2013; 34(3):519-529.

48. Papoutsi M, Labuschagne I, Tabrizi SJ, Stout JC. The cognitive burden in huntington's disease: Pathology, phenotype, and mechanisms of compensation. Mov Disord. 2014; 29(5):673-683.

49. Olesen PJ, Westerberg H, Klingberg T. Increased prefrontal and parietal activity after training of working memory. Nat Neurosci. 2004; 7(1):75-79.

50. Takeuchi H, Sekiguchi A, Taki Y, et al. Training of working memory impacts structural connectivity. J Neurosci. 2010; 30(9):3297-3303.

51. Nordvik JE, Schanke AK, Walhovd K, Fjell A, Grydeland H, Landro NI. Exploring the relationship between white matter microstructure and working memory functioning following stroke: A single case study of computerized cognitive training. Neurocase. 2012; 18(2):139-151.

52. Shoulson I, Fahn S. Huntington disease: Clinical care and evaluation. Neurology. 1979; 29(1):1-3.

53. Nasreddine ZS, Phillips NA, Bedirian V, et al. The montreal cognitive assessment, MoCA: A brief screening tool for mild cognitive impairment. J Am Geriatr Soc. 2005; 53(4):695-699.

54. Uttl B. North american adult reading test: Age norms, reliability, and validity. Journal of Clinical & Experimental Neuropsychology. 2002; 24(8):1123-1137.

55. Carlozzi NE, Stout JC, Mills JA, et al. Estimating premorbid IQ in the prodromal phase of a neurodegenerative disease. Clin Neuropsychol. 2011; 25(5):757-777.

56. Stout JC, Queller S, Baker KN, et al. HD-CAB: A cognitive assessment battery for clinical trials in huntington's disease 1,2,3. Mov Disord. 2014; 29(10):1281-1288.

57. Hocaoglu MB, Gaffan EA, Ho AK. The huntington's disease health-related quality of life questionnaire (HDQoL): A disease-specific measure of health-related quality of life. Clin Genet. 2012; 81(2):117-122.

58. Stern AF. The hospital anxiety and depression scale. Occup Med (Lond). 2014; 64(5):393-394.

59. Roth RM, Isquith PK, Gioia GA. Behavior Rating Inventory of Executive Function - Adult Version (BRIEF-A). Florida: Psychological Assessment Resources, Inc., 2005.

Appendix 1. List of outcome measures.

DOMAIN	Outcome measure	Description	Copyright / version date
Premorbid IQ	North American Adult Reading Test (NAART-35)[61]	Short version of the NAART (i.e. NAART-35). Shown to be reliable and valid in predicting WAIS-R Vocabulary scores. This test will be used to estimate premorbid IQ.	Uttl, 2002[61]
Criterion measures (i.e. tests similar to trained skills)	Digit Span -*Backward* (WMS-III)	Measure of auditory-verbal WM. The participant repeats a series of digits backwards.	Wechsler, 1997[74, 75]
	Spatial Span –*Forward* (WMS-III)	The examiner taps irregularly arranged cubes on a board in a non-sequential order. The patient is required to repeat the sequence of taps in forward order.	Wechsler, 1997[75]
Near transfer measures	Auditory-Verbal WM (WJ-III)	After hearing a series of digits and words, participants re-order the series, starting with the words and then digits in order.	Woodcock, McGrew, & Mather, 2001
	Spatial Span – *Backward* (WMS-III)	The examiner taps irregularly arranged cubes on a board in a non-sequential order. The patient is required to repeat the sequence of taps in backward order.	Wechsler, 1997[75]
Far transfer measures	Symbol Digits Modalities Test (SDMT; oral admin.)	Measure of visuo-perceptual scanning speed and attention. Score reflects the number of correct items produced in 90 seconds.	Smith, 1982[76]
	Phonetic verbal fluency (DKEFS)	Measure of phonemic fluency. Score reflects number of correct words produced across three 1-minute trials.	Delis, 2001[77]
	Trail Making Test (Part B)	Measure of psycho-motor speed, visuo-spatial attention, and flexibility.	Reitan, 1959[78]
	Hopkins Verbal Learning Test -revised.	Measure of verbal learning. Participant is required to recall a series of 12 words over three learning trials, free recall after a delay, and a recognition trial.	Brandt & Benedict, 2001[76]

Appendix 1 - continued

Questionnaires - outcomes			
**Mood**	Brief Symptom Inventory (BSI)	53-item questionnaire administered to assess behavioural and emotional symptoms, as well as their intensity at a specific point in time.	Derogatis, 1993
**Quality of life**	Huntington's Disease Quality of Life (HDQoL)	40 item questionnaire used to assess patient-reported outcomes related to health-related quality of life or the impact of health problems on personal well-being in people with HD.	Ho, Aileen
**Executive function**	Behavior Rating Inventory of Executive Function – Adult version (BRIEF-A)	Used to assess adult executive functioning and self-regulation	Roth, Isquith, Gioia, 2005 [70]
Questionnaires - for the assessment of individual characteristics			
**Leisure Activity**	Godin-Shepard Leisure Activity Questionnaire	Used to assess physical activity.	Godin, 1986
**Personality**	Revised Neo Personality Inventory (NEO PI-R); shortened version	Used to assess personality dimensions.	Costa & McCrae, 1992 [80]
**Motivation**	Situational Motivation Scale (SIMS)	Sixteen-item questionnaire used to assess the constructs of intrinsic motivation, identified regulation, external regulation, and amotivation.	Guay, Vallerand & Blanchard, 2000 [81]
**Socioeconomic Status**	Barratt Simplified Measure of Social Status	Measure of social status, which is a proxy for socioeconomic status. Score is based on educational level and occupational attainment (data collected in case history form)	Barratt, 2006[82]
